# Cold-Sintered ZnO Ceramic Composites Co-Doped with Polytetrafluoroethylene and Oxides

**DOI:** 10.3390/molecules29010129

**Published:** 2023-12-25

**Authors:** Yongjian Xiao, Yang Yang, Shenglin Kang, Yuchen Li, Xinyuan Hou, Chengjun Ren, Xilin Wang, Xuetong Zhao

**Affiliations:** 1 State Key Laboratory of Power Transmission Equipment Technology, Chongqing University, Shapingba District, Chongqing 400044, China; 202111021022@stu.cqu.edu.cn (Y.X.); 13595274397@163.com (Y.Y.); ksl124@cqu.edu.cn (S.K.); 202211021082t@stu.cqu.edu.cn (Y.L.); 202311021031@stu.cqu.edu.cn (X.H.); 2 Southwest Branch, State Grid Corporation of China, Chengdu 610041, China; gwxnfb@sw.sgcc.com.cn; 3 Tsinghua Shenzhen International Graduate School, Tsinghua University, Shenzhen 518055, China; wang.xilin@sz.tsinghua.edu.cn

**Keywords:** ceramic composite, cold sintering process, grain boundaries, ZnO

## Abstract

Grain boundaries play a significant role in determining the performance of ceramic-based materials. The modulation of interfacial structures provides a promising approach to improve the physicochemical and electrical properties of ceramic materials. In this work, the grain boundary structures of ZnO-based ceramics were manipulated by incorporating polytetrafluoroethylene (PTFE) and metal oxides through the cold sintering process (CSP). It was found that the grain size of ZnO-based ceramics can be effectively reduced from 525.93 nm to 338.08 nm with an addition of PTFE and metal oxides of CoO and Mn_2_O_3_. Microstructural results show that most of the PTFE phase and metal oxides were distributed along the grain boundaries, which may lead to the increased grain boundary resistance from 1.59 × 10^6^ ohm of pure ZnO to 6.21 × 10^10^ ohm of ZnO-based ceramics doped with PTFE and metal oxides, and enhanced Schottky barrier height from 0.32 eV to 0.59 eV. As a result, the breakdown field and nonlinear coefficient of the ZnO-based ceramics were improved to 3555.56 V/mm and 13.55, respectively. Therefore, this work indicates that CSP presents a feasible approach to design functional ceramic composites through the integration of polymer and metal oxides.

## 1. Introduction

Grain boundaries act as interfaces separating two homogeneous crystalline phases within polycrystalline systems such as ceramics, metals, or organic crystals [1,2,3]. The chemical properties and physical structures of the grain boundaries have significant impacts on the performances of both structural and functional materials [4,5]. Consequently, the manipulation of grain boundary structures was widely used to design new ceramic materials or improve their electrical and mechanical properties [6,7]. Specifically, for varistor ceramics with double Schottky barriers at the grain boundaries, the meticulous modulation and optimization of grain boundary structures significantly contributes to its electrical and physicochemical properties [8,9,10]. At present, a wide range of varistor materials such as ZnO, TiO_2_, SrTiO_3_, CaCu_3_Ti_4_O_12_, and SnO_2_ [11,12,13,14,15] have been extensively developed and studied. Among these materials, ZnO-based varistors are the most commonly employed as surge absorbers or lightning arrester in electronic circuits and power systems due to their excellent non-ohmic electrical behavior. The external lightning or internal switching of the circuit system may lead to transient overvoltage, which results in an abnormal current and damages the components used in the power system [16]. ZnO varistors, utilizing their non-ohmic current–voltage (*I*–*V*) characteristics, swiftly convert to a low-resistance state when the voltage exceeds the predesign threshold voltage, which can redirect overvoltage to the ground, preventing damage to the equipment and ensuring the stable operation of the circuit or system [16].

ZnO is a typical n-type semiconducting material with a wideband gap of 3.37 eV [17]. Generally, the stable ZnO presents a hexagonal crystal structure with a space group of C^4^_6v_ = *P*63mc [18], where O atoms are arranged in the hexagonal close-packed lattice. All octahedral voids and half of the tetrahedral voids are unoccupied by atoms. Therefore, the crystal structure of ZnO is in a relatively open state, making it easy for additive doping. The inherent ease for dopants introduced into ZnO makes it widely applied across various fields. Priyanka Jood et al. found that the incorporation of Ti and Al into ZnO can enhance the thermoelectric conversion capabilities of ZnO materials [19,20]. Dietl et al. reported that the introduction of magnetic ions into ZnO induces outstanding ferromagnetic properties [21]. Furthermore, as III-group elements such as Ga and In were doped into ZnO, a substantial increase in electrical conductivity can be achieved, broadening its applications in conductive oxides [22].

ZnO-based varistors were generally fabricated through the high-temperature sintering process of ZnO powder with various metal oxides such as Bi_2_O_3_, Co_2_O_3_, MnO_2_, Sb_2_O_3_, SiO_2_, etc. B_i2_O_3_, with a low melting point of 825 °C, is particularly important since it provides the medium for liquid-phase sintering, enhances the growth of ZnO grains, and affects the stability of the non-ohmic current–voltage characteristics of the material. The metal oxides were mainly distributed at the grain boundaries during the sintering and build a high Schottky barrier, which provides the basis for the non-ohmic behavior of ZnO-based ceramics [23]. The conventional sintering temperature of ZnO varistors is typically as high as around 1200 °C, with a long soaking time, spanning several hours to several days [24,25,26]. The high sintering temperature can induce an overgrowth of grains in ZnO varistor ceramics, resulting in a low-breakdown electrical field (*E*_b_) [27,28]. In order to mitigate the sintering temperature, many researchers have made significant efforts to develop novel low-temperature sintering techniques over the past decades such as liquid-phase sintering [29], microwave sintering [30], spark plasma sintering (SPS) [31], etc. Nevertheless, sintering temperatures of more than 800 °C are still required to complete the densification of ceramic materials. As a result, additives used for the grain boundary design of ZnO ceramics are limited to inorganic fillers only, since organic polymers can hardly resist temperatures above 400 °C. Traditionally, ceramic powders were homogeneously dispersed into a polymer matrix to produce a ceramic-polymer composites with an improved structure or electrical properties [32]. Regarding ceramic-based composites, ceramic powders are usually sintered first to form a ceramic skeleton, followed by the infiltration of polymers [33]. The differences in the processing temperature gap of ceramic and polymer materials make it difficult to fabricate ceramic-based composites without damaging the polymers in a single step.

In 2016, C. A. Randall et al. introduced the concept of the cold sintering process (CSP) to fabricate ceramic-based materials at a remarkably low temperature (≤300 °C) [34,35]. The cold sintering process covers several different mechanisms, and the critical enabling parameter is the transient chemical phase that activates the dissolution and precipitation process, together with a moderate temperature and pressure [36]. The densification process with CSP occurs under limited exposure to water vapor and/or aqueous solutions to provide a transient liquid phase that becomes evaporated under a uniaxial pressure with a controlled heating above 100 °C, in a die that is not completely closed. The CSP involves a series of interconnected nonequilibrium processes that collectively enable an irreversible densification of ceramic powders. First, the liquid phase aids particle rearrangement under the applied external pressure; the heating, pressure, and acidity accelerate chemical dissolution from powders to the aqueous liquid phase. With continued heating, this liquid evaporates, driving a high concentration of the solute, and eventually supersaturates the liquid and drives the precipitation and subsequent pseudo hydrothermal epitaxial crystal growth process that completes the final stages of sintering. The innovative sintering technology offers a solution to challenges that cannot be solved by conventional sintering processes and exhibits its potential applications in bridging the sintering gap of ceramics and polymers [37,38], reducing energy consumption and cost [39], and tailoring the grain size and grain boundaries [40] in ceramic materials and ceramic-based composites. Recently, ceramic–polymer composites were successfully produced via the CSP route. Zhao et al. [41] reported that ZnO–PTFE (polytetrafluoroethylene) composites could be designed based on CSP and densified at a low temperature of 285 °C. Furthermore, PTFE was also used to modulate the microstructure and electrical properties of other ceramics such as SiO_2_ [42] and BaTiO_3_ [43], based on CSP. Recently, Si et al. successfully integrated the thermoplastic polymer of poly-ether-ether–ketone (PEEK) with some nanoscale metal oxides into ZnO ceramic using CSP [5]. The ZnO–PEEK–oxide composite presents a switch-like effect which refers to high resistance state under a low electric field, and low resistance state under a high electric field.

Inspired by all these recent works, we prepared a new ZnO–PTFE–oxide (CoO, Mn_2_O_3_) composite employing CSP. Of the theoretical density of the ZnO-based composite, ~97% was achieved after an isothermal dwell at 300 °C for 1 h with an assisted pressure of 300 MPa. The effects of PTFE and metal oxides on the microstructural evolution of ZnO ceramics were observed via SEM. The breakdown electric field and non-linear coefficient of the ceramic composites were significantly improved. The roles of incorporating PTFE and metal oxides on the grain boundaries of the ceramic composites was discussed.

## 2. Results and Discussion

### 2.1. Density and Crystal Structure

**Figure 1:**
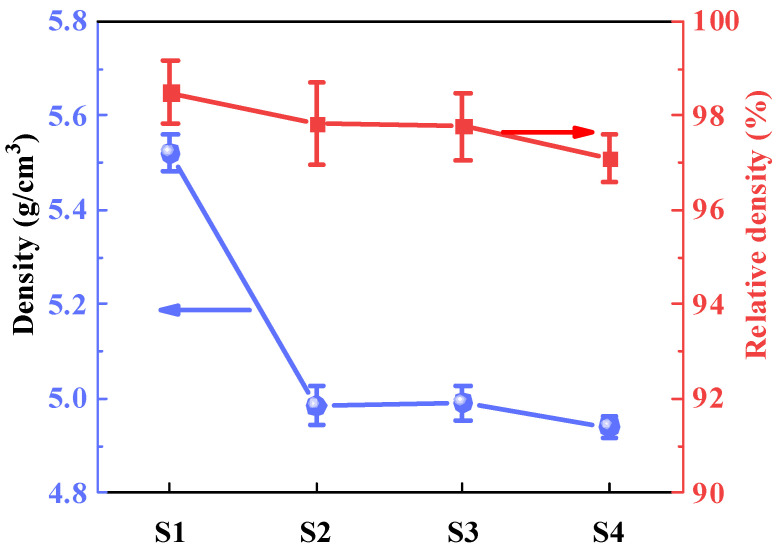
The density of ZnO–based composite ceramics.

Sintering parameters such as external pressure, sintering temperature, and soaking time are very crucial factors for ceramic densification during CSP. In 2017, S. Funahashi et al. [34] first prepared high-dense ZnO ceramics under 300 MPa at 305 °C using CSP. In this work, ZnO powder with particle size of ~200 nm was adopted, and its densification process was studied. As shown in Figures S1–S3 (Supplementary Materials), the various densities of ZnO ceramics fabricated under uniaxial pressure, from 50 MPa to 300 MPa, sintering temperature from room temperature (RT) to 300 °C, and soaking time from 0 to 9 h were studied using CSP. It was found that ZnO ceramics can be well densified when cold-sintered at 300 °C under a unixal presurre of 300 MPa for 1 h. These CSP parameters were also selected to fabricate the ZnO–PTFE composites. Figure 1 shows that all the samples present a high density of more than 97%. However, it is noteworthy that there is a sight downward trend in the relative density from samples S1 to S4, which can be associated with the increased presence of doping agents, mildly retarding the densification process.

**Figure 2:**
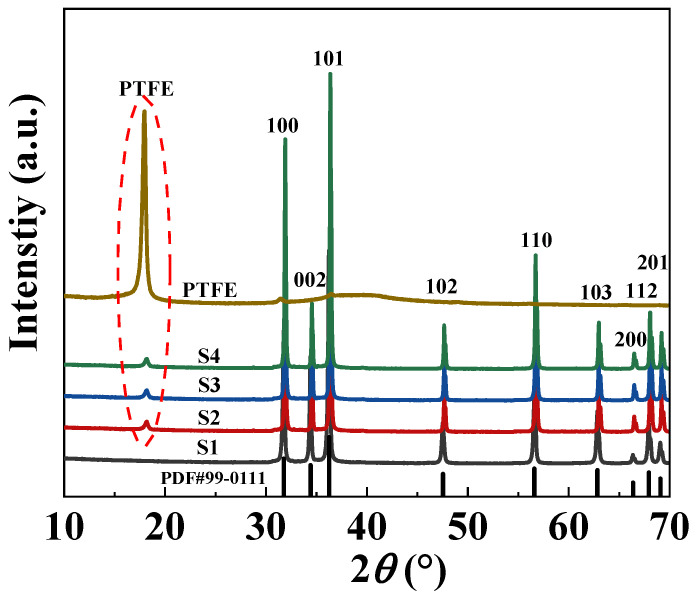
XRD patterns of ZnO-based composite ceramics.

Figure 2 demonstrates the X-ray diffraction (XRD) patterns of PTFE and cold-sintered ZnO samples S1, S2, S3, and S4. As a semi-crystalline polymer, PTFE has a combination of crystalline and amorphous structures and presents obvious X-ray peaks at around 18.0°. The major XRD peaks of S2, S3, and S4 are indexed with their Miller indexes based on the PDF#99–0111. Sample S1 presents the wurtzite structure with the space group of P63mc, and there are no obvious impurity X-ray peaks detected in ZnO ceramics, while for samples S2, S3, and S4, another very low peak at around 18.0° that can be attributed to the PTFE polymer can be found. However, the X-ray peaks of CoO and Mn_2_O_3_ doped in S3 and S4 were absent, which could have resulted from their low doping concentration or retention in the amorphous state during low-temperature CSP.

### 2.2. Microstructure and Dielectric Properties

**Figure 3:**
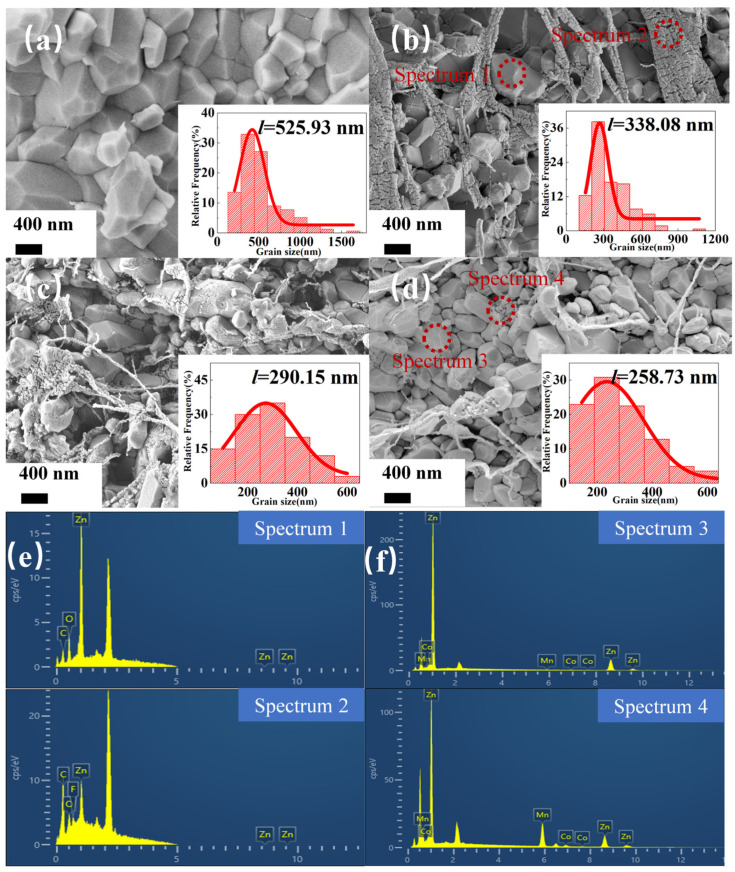
The microstructures of the fracture surfaces of samples sintered via CSP. The SEM images (**a**–**d**) for samples S1–S4 and the EDS elemental analysis spectra (**e**,**f**) for samples S2 and S4.

Figure 3a–d depicts the microstructural evolution of ZnO-based ceramic and composites with various additives. The mean grain size was calculated based on more than 100 grains using the linear intercepts method [44,45], as shown in the inset of Figure 3a–d. Figure 3a shows that the grain size of a pure ZnO ceramic increased from 200 nm of the initial powder to 525.93 nm after CSP. In Figure 3b, the grain size of the cold-sintered ZnO–PTFE composite was only 338.08 nm, suggesting that PTFE effectively inhibits excessive growth of the ZnO grain. Moreover, energy-dispersive spectroscopy (EDS) was employed to check the element distribution in sample S2, and two specific regions (Spectrum 1 and 2) were selected for point-scanning. Spectrum 1 was selected for ZnO grains, while Spectrum 2 was selected for the filamentous structures, as shown in Figure 3b. The elemental analysis spectra revealed that spectrum 1 exhibited a high content of Zn and O, whereas spectrum 2 displayed an obvious peak of the F element, corresponding to the polymer PTFE.

Figure 3c,d demonstrates the microstructure of samples S3 and S4, respectively. It can be observed that the grain size of the two samples decreased to 290.15 nm and 258.73 nm as CoO and Mn_2_O_3_ were introduced, indicating that the incorporation of metal oxides can further restrain grain growth. In Figure 3d, some segregated small particles can be observed at the grain boundaries and were proved to be Co and Mn elements according to the EDS results shown in Figure 3f. It can be inferred that CoO and Mn_2_O_3_ may not react with ZnO under CSP but preferentially segregate at the grain boundaries, thereby further suppressing grain growth.

**Figure 4:**
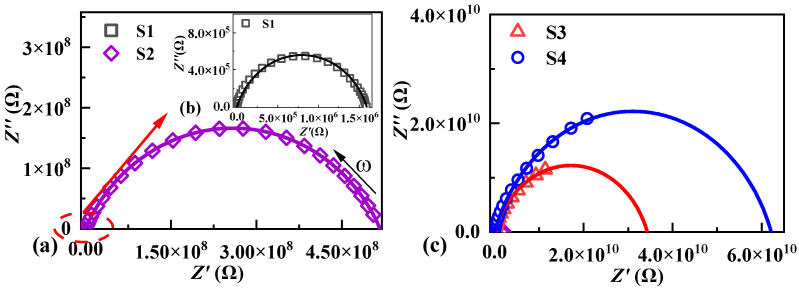
Nyquist plots of impedance for all samples at room temperature, (**a**) sample S2, (**b**) sample S1, (**c**) sample S3 and S4.

Figure 4 shows the impedance spectroscopy of samples S1, S2, S3, and S4 in the Nyquist plots at room temperature. The intercept at a low frequency corresponds to the resistance of the grain boundaries [46], which increases from 1.59 × 10^6^ ohm of sample S1 to 5.16 × 10^8^, 3.43 × 10^10^, and 6.21 × 10^10^ ohm with doping PTFE and metal oxides for sample S2, S3, and S4, respectively, while the high-frequency intercepts related to ZnO grain resistance remain nearly unchanged. The doped PTFE and metal oxides showed synergism in controlling the ZnO grain growth, meanwhile creating a high-resistance grain boundary phase between the smaller ZnO grains. Therefore, the incorporation of the PTFE and metal oxides CoO and Mn_2_O_3_ can dramatically increase the grain boundary resistance of ZnO ceramic composites, and improve the breakdown electric field of ZnO-based ceramic composites.

**Figure 5:**
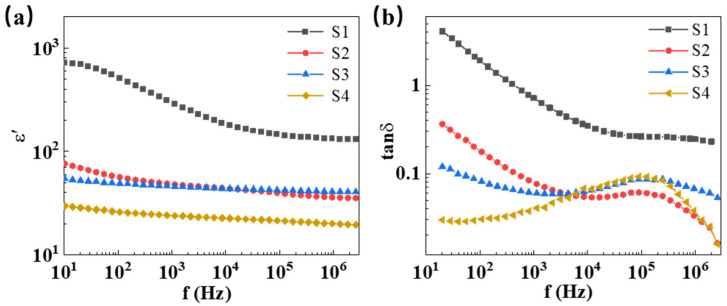
The permittivity (**a**) and dielectric losses (**b**) of samples at room temperature.

**Figure 6:**
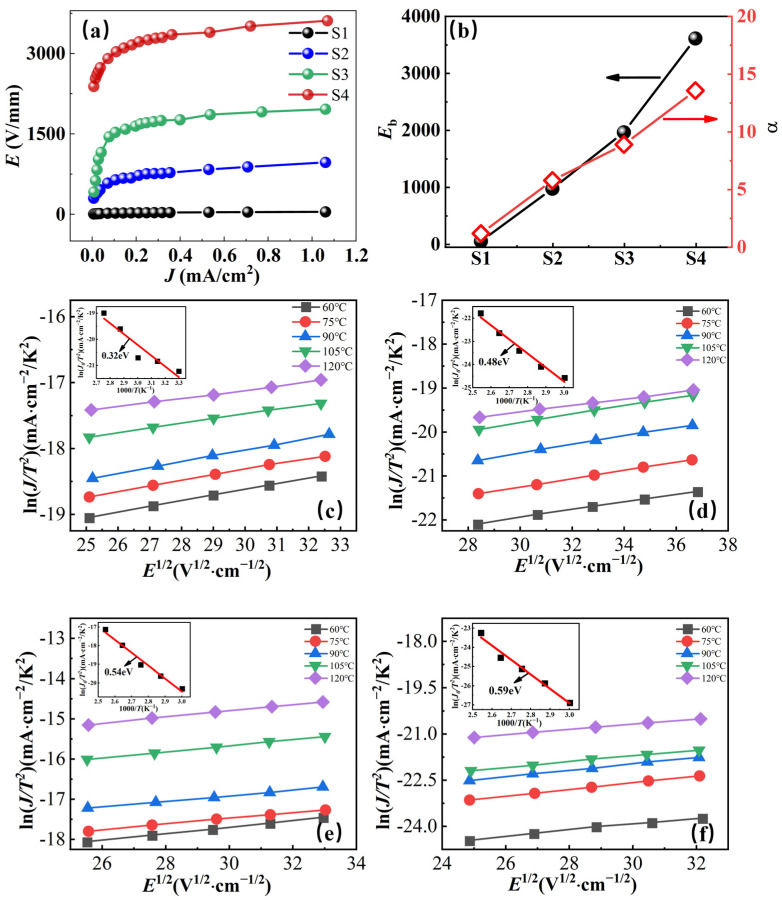
(**a**) Current-Voltage dependence of the ZnO-based varistor ceramics at room temperature. (**b**) The breakdown electric field (*E*_b_) and nonlinear coefficient (*α*) of ZnO-based varistor ceramics. Current-voltage-temperature data for ZnO-based varistor samples S1 (**c**), S2 (**d**), S3 (**e**), and S4 (**f**) in the temperature range of 60–120 °C. The Schottky barrier height is shown in the inset.

Figure 5a shows the frequency-dependent permittivity of ZnO-based ceramic composites at room temperature. It can be seen that the permittivity decreases with increasing frequency in the range from 10 Hz to 10^6^ Hz. Due to the low permittivity of PTFE (*ε_r_* = 2.1) [47], the permittivity of ZnO-based composites with PTFE is lower than that of pure ZnO ceramics. Furthermore, with the introduction of metal oxides CoO and Mn_2_O_3_, the permittivity of ZnO-based ceramic composites further decreases. In Figure 6b, the frequency-dependent variation in the dielectric loss tangent (tan*δ* = ε″/ε′) for ZnO-based ceramic composites were tested at room temperature. The relationship between dielectric loss tan*δ* and conductivity can be expressed as follows [48]:

(1) $$g=\frac{\varepsilon_{0}\left(\varepsilon_{s}-\varepsilon_{\infty }\right)\omega^{2}\tau }{1+\omega^{2}\tau^{2}}$$

(2) $$\tan\delta =\frac{\gamma +g}{\omega \varepsilon_{0}\varepsilon_{r}}$$

where *γ* is the dc conductivity of the dielectric, *g* is the equivalent conductivity of relaxation polarization, *τ* is relaxation time, *ω* is the angular frequency, *ε_s_* is the static relative permittivity, *ε*_∞_ is the optical frequency relative permittivity, *ε*_0_ is permittivity of the vacuum, and *ε_r_* is the relative permittivity of the dielectric. Equation (2) can be transformed into Equation (3) when *g* is much smaller than *γ* in the low-frequency range, and can be neglected as follows:

(3) $$\tan\delta =\frac{\gamma }{\omega \varepsilon_{0}\varepsilon_{r}}$$

where the predominant factor influencing the value of tan*δ* is the low-frequency dc conductivity.

As shown in Figure 5b, dielectric loss tan*δ* is inversely proportional to the frequency below 1000 Hz, indicating that dc conductance mainly contributes to dielectric loss in the low frequency range. Typically, at the power frequency of 50 Hz, sample S1 exhibits a very high dielectric loss tangent of 2.437, whereas the value of tan*δ* for sample S4 was significantly reduced to 0.028 because the contribution of dc conductivity to dielectric loss was well-suppressed with the introduction of PTFE and metal oxides. At high frequencies, a relaxation peak is observed in samples S2, S3, and S4, which can be attributed to the electronic relaxation processes of an intrinsic oxygen vacancy defect [49]. Thus, it is believed that incorporation of PTFE, Co, and Mn can optimize the grain boundary characteristics of ZnO-based ceramic composites, thereby enhancing their dielectric performance.

### 2.3. Nonlinear Ohmic Characteristics and Schottky Barriers

The *I*–*V* characteristics and nonlinear coefficient *α* of ZnO-based ceramic composites at room temperature are presented in Figure 6a,b, respectively. It can be found that the pure ZnO ceramic (S1) exhibits a quasi-ohmic behavior. As PTFE was introduced, *E*_b_ and *α* increased from 43 V/mm, 1.13 of sample S1 to 967 V/mm and 5.74 of sample S2. Moreover, with the introduction of metal oxides CoO and Mn_2_O_3_, *E*_b_ and *α* were further enhanced to 1960 V/mm, 8.86 of sample S3, and 3611 V/mm, 13.55 of sample S4, respectively.

In order to analyze the electrical conduction mechanism of cold-sintered ZnO-based ceramic composites, the current density–electric field (*J*–*E*) relationship at temperatures from 60 °C to 120 °C is given in Figure 6c,f. The *J*–*E* characteristics of ZnO-based ceramic composites follow the Schottky emission process and can be described by Equation (4) [41]:

(4) $$J=AT^{2}\exp[(\beta E^{1/2}-q\phi_{B})/kT]$$

where *J* is the current density, *T* is the absolute temperature, *A* is the Richardson constant, *E* is the applied electric field, *β* is a constant, k is the Boltzmann constant, and *Φ* is the Schottky barrier height. Under low electric fields, higher temperatures and electric field strength result in increased energy acquired by electrons. Consequently, electrons are more likely to overcome the barrier, leading to a higher current density. As shown in Figure 6c–f, the current density was apparently enhanced with the thermal excitation electric field *E* as the test temperature increased from 60 °C to 120 °C. On the other hand, a linear relationship between *E*^1/2^ and ln*J* was achieved, which indicates that the grain–grain boundary structures in ZnO-based ceramic composites are formed and consequently the thermionic emission current flows across the Schottky barrier in the grain boundary region as described by Equation (4). The Schottky barrier height of ZnO-based composite ceramics was determined using Arrhenius fitting (1/*T* and ln*J*/*T*^2^), as shown in the insets of Figure 6c–f. The barrier height gradually increased from 0.32 eV of sample S1 to 0.59 eV of sample S4.

From the *I*–*V* results, it is found that the introduction of polymer PTFE to ZnO ceramic induces a Schottky barrier of 0.48 eV at the grain boundary and results in the fundamental non-ohmic behavior of ZnO-PTFE composites [41]. The incorporation of CoO and Mn_2_O_3_ impedes grain growth and raises the Schottky barrier to 0.59 eV, enhancing the breakdown field and nonlinearity. It is believed that co-doping of PTFE and metal oxides in ZnO plays a synergistic effect in improving the *I*–*V* characteristics of ZnO-based ceramic composites.

## 3. Experimental Procedure

### 3.1. Sample Preparation

The initial ZnO powder with an approximate particle size of ~200 nm was purchased from Acros Organics (Morris Plains, NJ, USA). The PTFE suspension (60 wt%) dispersed in H_2_O was obtained from Sigma-Aldrich (Burlington, MA, USA), featuring an average particle size of ~0.5 microns. Mn_2_O_3_ powder was prepared by calcining Mn(OOCH_3_)_2_ (Alfa Aesar, Ward Hill, MA, USA) at 600 °C for 5 h. The CoO powder was acquired from Alfa Aesar with a purity of 99.9%.

**Figure 7:**
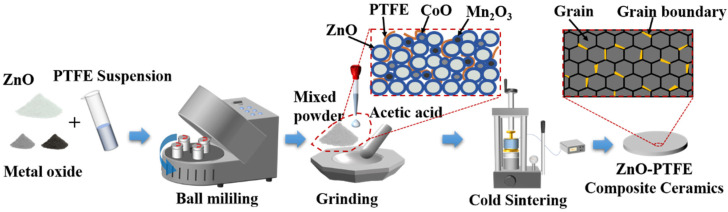
The schematic of the preparation of ZnO-based composites with PTFE and metal oxides using CSP.

As shown in Figure 7, ZnO powder was mixed with a PTFE suspension and two metal oxides (CoO and Mn_2_O_3_) based on the formulations presented in Table 1, and the initial composite powders denoted as P1, P2, P3, and P4 were prepared, respectively. The ball milling equipment with a model of XQM-2 was purchased from Changsha Tianchuang Powder Technology Co., Ltd. (Changyuan, China). The rotation speed during the ball milling process was set to 380 revolutions per minute. Then, the resulting mixtures were dried in an oven at 70 °C for 12 h. Afterward, a small quantity of an acetic acid solution (2 mol/L, approximately 20 wt%) was introduced to the mixture and manually ground in a mortar for a few minutes. Then, the moistened powders were poured into a steel die with a diameter of 12.7 mm, and pressed using a manual press (model YLJ-24T) under a pressure of 300 MPa, first at room temperature for 10 min, and then the steel die was heated to 300 °C with a ramp rate of 15 °C per minute. The temperature was isothermally kept at 300 °C for 1 h, and then cooled down to room temperature before taking out the sample. The ZnO ceramic composites prepared from the four different initial powders (P1, P2, P3, and P4) were labeled as S1, S2, S3, and S4, respectively.

### 3.2. Characterization

The relative densities of the cold sintered samples were measured using a geometric and an Archimedes method in ethanol. In the experimental procedure, the samples underwent polishing and ultrasonic cleaning in anhydrous ethanol to eliminate surface impurities. Subsequently, a constant-temperature drying oven (DHG-9013A) was employed to remove any residual liquid from the samples. The completely dried samples were then weighed on a precision analytical balance, resulting in a recorded mass denoted as *m*_1_ (g). Subsequently, the samples were immersed in anhydrous ethanol and placed on a filter, with the measured mass recorded as *m*_2_ (g). After the removal of anhydrous ethanol and surface drying, the saturated weight, *m*_3_ (g), was determined. Using the density of anhydrous ethanol (*ρ*_w_), the real density of the ceramic samples (*ρ*) was calculated using Equation (5). The relative density was then calculated based on the theoretical density (*ρ*′), as shown in Equation (6).

(5) $$\rho =\frac{m_{1}}{m_{3}-m_{2}}\times \rho_{w}$$

(6) $$\rho_{r}=\frac{\rho }{\rho^{\prime }}\times 100\%$$

X-ray diffraction analysis (PANalytical Empyrean, Stanford, CA, USA) was employed for a comprehensive assessment of the phase composition of the samples, utilizing Cu-Kα radiation with a wavelength (λ) of 0.15416 nm. The scanning range of 2-theta angles spanned 10° to 70°, with a step size of 0.026°, enabling the acquisition of detailed crystalline information. Microstructural observations of the cross-sections of the ZnO-based composite ceramic samples were conducted using a field emission scanning electron microscope (SEM, JEOL JSM-6390A, Tokyo, Japan) to characterize the morphology and investigate the influence of additive doping on the internal grain boundary structure, with magnifications ranging from 20,000 to 50,000 times. The structural features and distribution of dopants were determined through point scanning tests using energy-dispersive spectroscopy (EDS). For the electrical measurement, both sides of the samples were polished and sputtered with Au as electrodes by a sputter coater (Quorum Q150T). The diameters of the electrodes on both sides of the samples were 8 mm and 6 mm, respectively, which were both smaller than the diameter (12.7 mm) of the samples to prevent the surface–flashover breakdown during the current density–electric field property test.

The current density–electric field characteristics were measured using a pA meter (HP4140B, Hewlett Packard, Palo Alto, CA, USA) and the temperature was controlled by a Delta oven (Delta 9023, Cohu Inc., Poway, CA, USA) in the range of room temperature to 120 °C. The breakdown field, *E*_b_, was determined using Equation (7) and the nonlinear coefficient *α* was obtained via Equation (8), where *d* is the thickness of the sample, and *U*_1mA_ and *U*_0.1mA_ are the voltage measured at the current of 1.0 mA and 0.1 mA, respectively. For the testing and analysis of the barrier heights, it was necessary to measure the *J*–*E* characteristics at different temperatures. The samples fixed in an electrode system were placed in a Delta oven for the temperature–variable *J*–*E* test. Impedance spectra and dielectric loss were tested via broadband dielectric spectroscopy (Novocontrol Concept 80, Montabaur, Germany) using a 1 V peak-to-peak ac small-signal voltage in the range from 10 Hz to 10^6^ Hz.

(7) $$E_{b}=\frac{U_{1\mathrm{mA}}}{d}$$

(8) $$\alpha =\frac{\log_{10}(I_{1\mathrm{mA}}/I_{0.1\mathrm{mA}})}{\log_{10}(U_{1\mathrm{mA}}/U_{0.1\mathrm{mA}})}=\frac{1}{\log_{10}(U_{1\mathrm{mA}}/U_{0.1\mathrm{mA}})}$$

## 4. Conclusions

In summary, a new type of ZnO–PTFE–oxide ceramic composite was introduced, which presented an extremely high-breakdown electrical field and improved nonlinear coefficient. The ZnO-based ceramic composites were fabricated using CSP at 300 °C under a uniaxial pressure of 300 MPa, and achieved a large relative density of ≥97%. The microstructural results show that the grain sizes of ZnO were severely restricted to hundreds of nanometers, and filamentous PTFE and segregated metal oxides could be found at the grain boundaries. The introduction of PTFE and metal oxide fillers to the ZnO ceramic during CSP synergistically produced a high-resistance grain boundary phase, which induced the non-ohmic electrical behavior and enhanced the breakdown field. Therefore, the varistor-like *J*–*E* behavior of the ZnO–PTFE–oxide ceramic composite was observed due to the Schottky barrier at the grain boundaries. The impedance spectroscopy analysis indicated that the contribution of dc conductivity to dielectric loss can be restrained because the incorporation of the PTFE phase and metal oxides increased the Schottky barrier height at the grain boundaries. This work provides a promising processing route for grain boundary design with polymer and metal oxides in ceramic composites through the cold sintering process.

## Figures and Tables

**Table 1:** Various composite powders (mol%) of ZnO–PTFE–oxides.

Composite Powder	ZnO	PTFE	CoO	Mn_2_O_3_
P1	100	0	0	0
P2	85	15	0	0
P3	84	15	1	0
P4	83.5	15	1	0.5

## Data Availability

The data presented in this study are available in article.

## Supplementary Materials

The following supporting information can be downloaded at: https://www.mdpi.com/article/10.3390/molecules29010129/s1, Figure S1. The density of ZnO ceramic samples at different sintering pressures. Figure S2. The density of ZnO ceramic samples at different sintering temperatures. Figure S3. The density of ZnO ceramic samples at different sintering time.

## References

[1] Żydek A., Wermiński M., Trybula M.E. (2021). Description of Grain Boundary Structure and Topology in Nanocrystalline Aluminum Using Voronoi Analysis and Order Parameter. Comput. Mater. Sci..

[2] Chen I.W., Wang X.H. (2000). Sintering Dense Nanocrystalline Ceramics without Final-Stage Grain Growth. Nature.

[3] Van Swygenhoven H., Weertman J.R. (2006). Deformation in Nanocrystalline Metals. Mater. Today.

[4] Vladimirov I., Kühn M., Geßner T., May F., Weitz R.T. (2018). Energy Barriers at Grain Boundaries Dominate Charge Carrier Transport in an Electron-Conductive Organic Semiconductor. Sci. Rep..

[5] Si M., Guo J., Hao J., Zhao X., Randall C.A., Wang H. (2021). Cold Sintered Composites Consisting of PEEK and Metal Oxides with Improved Electrical Properties via the Hybrid Interfaces. Compos. Part B Eng..

[6] Chen J., Park N.G. (2020). Materials and Methods for Interface Engineering toward Stable and Efficient Perovskite Solar Cells. ACS Energy Lett..

[7] Zhang X., Hu T., Rufner J.F., LaGrange T.B., Campbell G.H., Lavernia E.J., Schoenung J.M., van Benthem K. (2015). Metal/Ceramic Interface Structures and Segregation Behavior in Aluminum-Based Composites. Acta Mater..

[8] Gupta T.K. (1990). Application of Zinc Oxide Varistors. J. Am. Ceram. Soc..

[9] Greuter F., Blatter G. (1990). Electrical Properties of Grain Boundaries in Polycrystalline Compound Semiconductors. Semicond. Sci. Technol..

[10] Recnik A., Bernik S., Daneu N. (2012). Microstructural Engineering of ZnO-Based Varistor Ceramics. J. Mater. Sci..

[11] Masteghin M.G., Varela J.A., Orlandi M.O. (2017). Controlling the Breakdown Electric Field in SnO_2_ Based Varistors by the Insertion of SnO_2_ Nanobelts. J. Eur. Ceram. Soc..

[12] Staykov A., Tellez H., Druce J., Wu J., Ishihara T., Kilner J. (2018). Electronic Properties and Surface Reactivity of SrO-Terminated SrTiO_3_ and SrO-Terminated Iron-Doped SrTiO_3_. Sci. Technol. Adv. Mater..

[13] Liang J., Zhao X., Sun J., Ren L., Liao R., Yang L., Li W. (2020). Enhanced Electrical Properties of ZnO Varistor Ceramics by Spark Plasma Sintering: Role of Annealing. Ceram. Int..

[14] Yan M.F., Rhodes W.W. (1982). Preparation and Properties of TiO_2_ Varistors. Appl. Phys. Lett..

[15] Tang Z., Wu K., Li J., Huang S. (2020). Optimized Dual-Function Varistor-Capacitor Ceramics of Core-Shell Structured xBi_2/3_Cu_3_Ti_4_O_12_/(1-x)CaCu_3_Ti_4_O_12_ Composites. J. Eur. Ceram. Soc..

[16] Tian T., Zheng L., Podlogar M., Zeng H., Bernik S., Xu K., Ruan X., Shi X., Li G. (2021). Novel Ultrahigh-Performance ZnO-Based Varistor Ceramics. ACS Appl. Mater. Interfaces.

[17] Dillip G.R., Banerjee A.N., Joo S.W. (2020). Template-Based Synthesis of Hollow Nanotubular ZnO Structures and Nonlinear Electrical Properties under Field-Induced Trap-Assisted Tunneling. J. Phys. Chem. C.

[18] Triboulet R., Perrière J. (2003). Epitaxial Growth of ZnO Films. Prog. Cryst. Growth Charact. Mater..

[19] Jood P., Mehta R.J., Zhang Y., Peleckis G., Wang X., Siegel R.W., Borca-Tasciuc T., Dou S.X., Ramanath G. (2011). Al-Doped Zinc Oxide Nanocomposites with Enhanced Thermoelectric Properties. Nano Lett..

[20] Cai K.F., Müller E., Drašar C., Mrotzek A. (2003). Preparation and Thermoelectric Properties of Al-Doped ZnO Ceramics. Mater. Sci. Eng. B.

[21] Dietl T., Ohno H., Matsukura F., Cibert J., Ferrand D. (2000). Zener Model Description of Ferromagnetism in Zinc-Blende Magnetic Semiconductors. Science.

[22] Shen H., Zhang H., Lu L., Jiang F., Yang C. (2010). Preparation and Properties of AZO Thin Films on Different Substrates. Prog. Nat. Sci. Mater. Int..

[23] Clarke D.R. (1999). Varistor ceramics. J. Am. Ceram. Soc..

[24] Sato Y., Yamamoto T., Ikuhara Y. (2007). Atomic Structures and Electrical Properties of ZnO Grain Boundaries. J. Am. Ceram. Soc..

[25] Matsuoka M. (1971). Nonohmic Properties of Zinc Oxide Ceramics. Jpn. J. Appl. Phys..

[26] Hembram K., Rao T.N., Srinivasa R.S., Kulkarni A.R. (2015). High Performance Varistors Prepared from Doped ZnO Nanopowders Made by Pilot-Scale Flame Spray Pyrolyzer: Sintering, Microstructure and Properties. J. Eur. Ceram. Soc..

[27] Daneu N., Rečnik A., Bernik S. (2011). Grain-Growth Phenomena in ZnO Ceramics in the Presence of Inversion Boundaries. J. Am. Ceram. Soc..

[28] He J. (2019). Metal Oxide Varistors: From Microstructure to Macro-Characteristics.

[29] Bernik S. (2018). Low-temperature sintering of ZnO–Bi_2_O_3_-based varistor ceramics for enhanced microstructure development and current-voltage characteristics. Ceram.–Silik..

[30] Subasri R., Asha M., Hembram K., Rao G.V.N., Rao T.N. (2009). Microwave Sintering of Doped Nanocrystalline ZnO and Characterization for Varistor Applications. Mater. Chem. Phys..

[31] Macary L.S., Kahn M.L., Estournès C., Fau P., Trémouilles D., Bafleur M., Renaud P., Chaudret B. (2009). Size Effect on Properties of Varistors Made from Zinc Oxide Nanoparticles through Low Temperature Spark Plasma Sintering. Adv. Funct. Mater..

[32] Arbatti M., Shan X., Cheng Z.-Y. (2007). Ceramic–Polymer Composites with High Dielectric Constant. Adv. Mater..

[33] Yuan S., Shen F., Chua C.K., Zhou K. (2019). Polymeric Composites for Powder-Based Additive Manufacturing: Materials and Applications. Prog. Polym. Sci..

[34] Funahashi S., Guo J., Guo H., Wang K., Baker A.L., Shiratsuyu K., Randall C.A. (2017). Demonstration of the Cold Sintering Process Study for the Densification and Grain Growth of ZnO Ceramics. J. Am. Ceram. Soc..

[35] Guo J., Guo H., Baker A.L., Lanagan M.T., Kupp E.R., Messing G.L., Randall C.A. (2016). Cold Sintering: A Paradigm Shift for Processing and Integration of Ceramics. Angew. Chem..

[36] Guo J., Floyd R., Lowum S., Maria J.-P., Herisson de Beauvoir T., Seo J.-H., Randall C.A. (2019). Cold Sintering: Progress, Challenges, and Future Opportunities. Annu. Rev. Mater. Res..

[37] Guo J., Berbano S.S., Guo H., Baker A.L., Lanagan M.T., Randall C.A. (2016). Cold Sintering Process of Composites: Bridging the Processing Temperature Gap of Ceramic and Polymer Materials. Adv. Funct. Mater..

[38] Guo J., Zhao X., Herisson De Beauvoir T., Seo J.-H., Berbano S.S., Baker A.L., Azina C., Randall C.A. (2018). Recent Progress in Applications of the Cold Sintering Process for Ceramic–Polymer Composites. Adv. Funct. Mater..

[39] Sohrabi Baba Heidary D., Lanagan M., Randall C.A. (2018). Contrasting Energy Efficiency in Various Ceramic Sintering Processes. J. Eur. Ceram. Soc..

[40] Zhao X., Liang J., Sun J., Guo J., Dursun S., Wang K., Randall C.A. (2021). Cold Sintering ZnO Based Varistor Ceramics with Controlled Grain Growth to Realize Superior Breakdown Electric Field. J. Eur. Ceram. Soc..

[41] Zhao X., Guo J., Wang K., Herisson De Beauvoir T., Li B., Randall C.A. (2018). Introducing a ZnO–PTFE (Polymer) Nanocomposite Varistor via the Cold Sintering Process. Adv. Eng. Mater..

[42] Ndayishimiye A., Tsuji K., Wang K., Bang S.H., Randall C.A. (2019). Sintering Mechanisms and Dielectric Properties of Cold Sintered (1-x) SiO_2_—x PTFE Composites. J. Eur. Ceram. Soc..

[43] Sada T., Tsuji K., Ndayishimiye A., Fan Z., Fujioka Y., Randall C.A. (2021). High Permittivity BaTiO_3_ and BaTiO_3_-Polymer Nanocomposites Enabled by Cold Sintering with a New Transient Chemistry: Ba(OH)_2_∙8H_2_O. J. Eur. Ceram. Soc..

[44] Wurst J.C., Nelson J.A. (1972). Lineal Intercept Technique for Measuring Grain Size in Two-Phase Polycrystalline Ceramics. J. Am. Ceram. Soc..

[45] Zhao X., Xiao Y., Kang S., Li Y., Cheng L., Ren C., Guo J., Wang X., Yang L., Liao R. (2023). Comparative Study on Energy Efficiency and Densification of ZnO Ceramics Using Various Sintering Processes. J. Mater. Sci. Mater. Electron..

[46] Sinclair D.C., West A.R. (1989). Impedance and Modulus Spectroscopy of Semiconducting BaTiO_3_ Showing Positive Temperature Coefficient of Resistance. J. Appl. Phys..

[47] Xiang F., Wang H., Yao X. (2006). Preparation and Dielectric Properties of Bismuth-Based Dielectric/PTFE Microwave Composites. J. Eur. Ceram. Soc..

[48] Zhao X., Li J., Li H., Li S. (2012). Intrinsic and Extrinsic Defect Relaxation Behavior of ZnO Ceramics. J. Appl. Phys..

[49] Zhao X., Liao R., Liang N., Yang L., Li J., Li J. (2014). Role of Defects in Determining the Electrical Properties of ZnO Ceramics. J. Appl. Phys..

