# Supplementary material for: Cold-Sintered ZnO Ceramic Composites Co-Doped with Polytetrafluoroethylene and Oxides
Source: Molecules. 2023 Dec 25;29(1):129. doi: 10.3390/molecules29010129 (PMC10779997; doi:10.3390/molecules29010129)
Supplement: Supplementary file 1 [file molecules-29-00129-s001.zip › molecules-2773469-supplementary.pdf]

Supplementary Materials

Cold sintered ZnO ceramic composites co-doped with polytetrafluoroethylene and oxides

Yongjian Xiao [1], Yang Yang [1], Shenglin Kang [1], Yuchen Li [1], Xinyuan Hou [1], Chengjun Ren [2], Xilin Wang [3], Xuetong Zhao [1,*]

[1] State Key Laboratory of Power Transmission Equipment & System Security and New Technology, Chongqing University, Shapingba District, Chongqing, 400044, PR China; 202111021022@stu.cqu.edu.cn (Y.X.); 985232908@qq.com (Y.Y.); ksl124@cqu.edu.cn (S.K.); 202211021082t@stu.cqu.edu.cn (Y.L.); 202311021031@stu.cqu.edu.cn (X.H.);
[2] Southwest Branch, State Grid Corporation of China, Chengdu 610041, Sichuan, People's Republic of China; gwxnfb@sw.sgcc.com.cn (C.R.);
[3] Tsinghua Shenzhen International Graduate School, Tsinghua University, Shenzhen 518055, Guangdong, China; wang.xilin@sz.tsinghua.edu.cn (X.W.);
* Correspondence: zxt201314@cqu.edu.cn (X.Z.);

Supplementary Figure

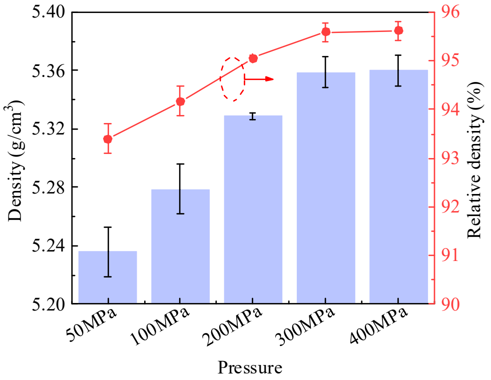

Figure S1. The density of ZnO ceramic samples at different sintering pressures

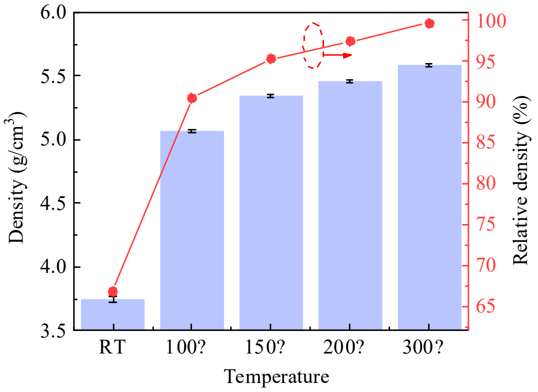

Figure S2. The density of ZnO ceramic samples at different sintering temperatures

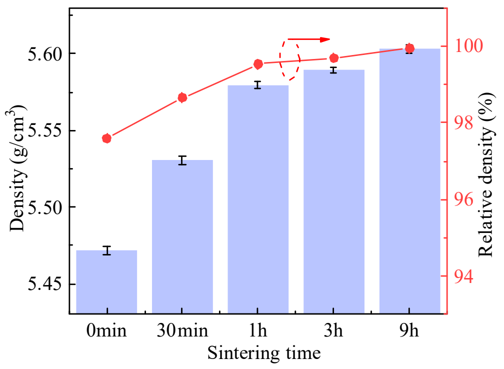

Figure S3. The density of ZnO ceramic samples at different sintering time
